# Depressive symptoms are more influenced by personality traits and styles than working in nursing—a study during the COVID-19 pandemic

**DOI:** 10.3389/fpsyg.2023.1138185

**Published:** 2023-08-21

**Authors:** Victoria Elena Maringgele, Martin Scherr, Wolfgang Aichhorn, Andreas Kurt Kaiser

**Affiliations:** Department of Psychiatry, Psychotherapy and Psychosomatics, Christian Doppler Medical Center, Paracelsus Medical University, Salzburg, Austria

**Keywords:** COVID-19 pandemic, nurses, personality, well-being, depression

## Abstract

**Background:**

According to literature, the COVID-19 pandemic caused stressful working conditions for nurses, which may have a negative impact on their Well-Being and mental health.

**Aim:**

To investigate whether nurses and *non*-helping professionals differ in their Well-Being. Furthermore, we analyzed, for the first time, which personality traits and styles are a risk factor for nurses’ wellbeing during COVID-19 pandemic.

**Methods:**

In an online survey, the following psychological tests were used on nursing staff (*n* = 518) and *non*-helping professionals (*n* = 335): *WHO-Five* (*WHO-5*), the *Personality, Style and Disorder Inventory* (*PSSI*), and the *Freiburg Personality Inventory-Revised* (*FPI-R*).

**Results:**

Nurses and *non*-helping professionals did not differ significantly in terms of Well-Being. The Well-Being of nurses was correlated with the following personality traits and styles, namely *Spontaneous-Borderline Personality Style*, *Silent-Depressive Personality Style*, *Strain*, *Emotionality*, and *Life Satisfaction*. According to our results, 33% of participants suffered from clinically significant depressive symptoms.

**Discussion:**

According to our results, nurses are not more at risk for depression. However, it was shown that Well-Being during the pandemic is highly dependent on personality.

**Conclusion:**

Specific personality traits and styles are a greater predictor of depressive symptoms than profession. The stressful occupational environment during COVID-19 pandemic is not the only cause for depressive symptoms in nurses. Psychotherapeutic interventions are especially important for particular individuals and are necessary to prevent depressive symptoms during COVID-19 pandemic.

## Introduction

Studies have shown negative effects of the COVID-19 pandemic, such as that COVID-19 patients were burdened by depression, anxiety disorders, stress, and panic attacks (Hossain et al., 2020). According to Nobari et al. (2021), COVID-19 also negatively affects the quality of life of children and adolescents. Amerio et al. (2021) demonstrated that after COVID-19 blocking, the prevalence of depressive and anxiety symptoms, sleep disturbances, and poor quality of life increased significantly in adults. An association between these symptoms and gender, smoking, and physical activity was demonstrated (Amerio et al., 2021). In addition, psychotropic medication use increased during COVID-19 (Amerio et al., 2021). Ambrosetti et al. (2021) also demonstrated the negative impact of the pandemic on psychiatric patients.

During the last 3 years, healthcare workers and, especially nurses caring for patients suffering from coronavirus disease 2019 (Sars-Cov-2), have been exposed to increased workloads due to the COVID-19 pandemic (Riedel et al., 2021). In addition, nurses are the group of healthcare workers most commonly infected with COVID-19 (Gómez-Ochoa et al., 2021). According to recent publications, the stressful work and life situation of nurses had a negative impact on their Well-Being (Ashley et al., 2021; Yayla and Eskici, 2021), mental health, and work-life balance (Yayla and Eskici, 2021). Recent studies also showed that nurses are at high risk of burnout during the COVID-19 pandemic (Galanis et al., 2021) and that some nurses even developed what is known as “coronaphobia” (Yayla and Eskici, 2021). Also, a large proportion of those responsible for COVID-19 patients reported symptoms, such as mental distress, depression, thoughts of self-harm, anxiety and insomnia, even suicidal ideation, and suicide-attempts (Chidiebere et al., 2020; Lai et al., 2020; Galanis et al., 2021; Li et al., 2021; Marcomini et al., 2021; Sriharan et al., 2021; Varghese et al., 2021). It is unclear whether certain personality traits of nurses act as a protective factor or risk factor for mental health. Possibly Well-Being during COVID-19 pandemic depends not only on external circumstances (Labrague and De Los, 2020), but also on personality traits and styles.

Nédélec et al. (2021) suggest that examining personality profile can be useful to identify higher risk for stress sensitivity. According to Lecic-Tosevski et al. (2011), a resilient personality is one that has the ability to cope with stressful situations. In turn, good resilience provides a sense of Well-Being (Babić et al., 2020). Other findings showed that personality traits are related to vulnerability to anticipatory stress and stress management effectiveness (Schlatter et al., 2022). A study of nurses’ personality and stress found that nurses with strong type A personality and high levels of neuroticism suffered more stress (Lu et al., 2022). Mason et al. (2020) claimed, based on their study results, that nurses with higher levels of neuroticism and lower levels of extraversion are less suited to their jobs.

### Aim and hypotheses of study

Identifying risk factors for psychological distress is important for nurses and the health care system (Galanis et al., 2021), especially during the challenging COVID-19 pandemic. Because personality is one of the most important predictors of subjective Well-Being (Serrano et al., 2020), we examined whether personality traits influence Well-Being during the COVID-19 pandemic. Based on the stressful life situations just described during the pandemic, we hypothesized that nurses and non-nursing professionals would differ statistically significantly in their Well-Being. It was expected that nurses would have lower Well-Being than the control group because of their working conditions. Comparing nurses’ Well-Being to that of non-nurses could shed light on whether nurses’ Well-Being is more affected during a pandemic. Another goal of this study was to shed light on the influence of nurses’ low Well-Being and whether it was more influenced by personality or workplace during COVID-19. These findings should also inform the development of protective measures for individuals with personality.

## Methods

### Sample

Overall, 878 participants completed three questionnaires. People between the ages of 18 and 69 were included in the study. 25 participants were excluded from the study for a variety of reasons: Some participants were excluded because they were either psychologists, psychotherapists, or physicians were excluded because they work in a helping profession, although they are not nurses. Study nurses, teachers at the nursing school, nurses in education, nurses working at the Nursing Directorate, and surgical assistants were also excluded because they did not work directly with patients, and their contact to the subject under consideration may produce bias. People in training were excluded from both groups too because only those currently working or those who had worked during the pandemic were interviewed. 853 participants in total, including 518 nursing staff and 335 individually in a non-helping profession in the control group, were enrolled in the analysis. The mean age of the main group (nursing staff, caring for patients infected with COVID-19 disease) and the mean age of the control group (people of *non*-helping professions) did not differ statistically significantly (*p* = 0.507). The mean (*M*) age of nursing staff was 39.32 (18–69) and the mean age of *non*-helping professionals was 39.84 (19–64).

Gender characteristics of the participants are shown in Table 1. Furthermore age, sex, relationship status, extent of employment, and duration of employment were recorded. With regard to data protection, no additional information was collected about our participants. The study was performed between April 2021 until May 2022, during the peak of the COVID-19 pandemic and the time of rapid and new development of new SARS-CoV-2 virus variants: alpha, delta, and omicron. Participants were recruited at a large public hospital, especially at the Salzburg state clinic (University Hospital Salzburg, Christian-Doppler-Klinik), Red Cross, and other companies. The Bundesministerium für Gesundheit [The Federal Ministry of Health] (2021) calculated that 84% of nursing staff are female which reflected the gender distribution in the nursing cohort in our study (approximately 80% female). The survey was created using LimeSurvey program (LimeSurvey GmbH, 2020). Participants received a link and filled out the questionnaires online after giving their written informed consent. The study was approved by the Ethics Commission of the State of Salzburg.

**Table 1:** Gender characteristics of participants.

	Nurses (*n* = 518)		Controls (*n* = 335)	
	*n*	%	*n*	%
Female	415	80	205	61
Male	103	20	130	39

Sample size = *n*.

### Measuring instruments

Within a year of the start of the COVID-19 pandemic, participants’ wellbeing as well as their personality traits and styles were examined, using the following psychological test procedures.

The psychological test procedure Personality, Style and Disorder Inventory (PSSI) is a self-assessment instrument, recording 14 personality styles, namely Willful-Paranoid Personality Style (PS), Reserved-Schizoid PS, Foreboding-Schizotypical PS, Spontaneous-Borderline PS, Amiable-Histrionic PS, Ambitious-Narcissistic PS, Self-Critical-Self-Insecure PS, Loyal-Dependent PS, Carefully-Obsessive–Compulsive P, Critical-negativistic PS, Silent-Depressive PS, Helpful-Selfless PS, Optimistic-Rhapsodic PS, and Assertive-Antisocial PS (Kuhl and Kazén, 2009). The raw scores were used for statistical analysis. Cronbach’s alpha of the PSSI scales vary between *α* = 0.73 and 0.85. Good construct validity of the inventory was also demonstrated (Kuhl and Kazén, 2009).

The *Freiburg Personality Inventory, Revised* (*FPI-R*) records personality traits. The tested personality traits were selected by the authors from different theories and approaches. It consists of 138 items and 12 scales, namely Life Satisfaction, Social Orientation, Achievement Orientation, Inhibitedness, Impulsiveness, Aggressivenes, Strain, Somatic Complaints, Health Concern, Frankness, Extraversion, and Emotionality (Fahrenberg et al., 2010). The raw scores were used for statistical analysis. Cronbach’s alpha of the scales is between *α* = 0.73 and *α* = 0.83 and the empirical validity is proven (Fahrenberg et al., 2010).

The *WHO-Five Well-Being Index* (*WHO-5*) is a screening questionnaire to record Well-Being. The advantages of the *WHO-5* are its brevity and validity as a screening tool for depression (Topp et al., 2015). According to Brähler et al. (2007), the German version of the *WHO-5* has a very good psychometric performance. This screening consists of five questions. The raw score ranges from 0 to 25, with 0 denoting the lowest Well-Being and 25 denoting the greatest Well-Being. A value of 13 is the critical limit: Depressive symptoms can be assumed with scores below this value (Index der Weltgesundheitsorganisation zum Wohlbefinden WHO-5, 2022).

### Statistical methods

The calculations were performed using SPSS v.27 (IBM Statistics SPSS, 2021). A *Pearson correlation* and a *t-test for independent samples* were used for calculations. In addition to the *t-*test, the following other methods were used: The *Shapiro–Wilk test* was used to check whether the group data were normally distributed. In addition, the *Levene test* was calculated to check the homogeneity of the variance. The hypotheses were checked for two-sided significance. A total of 28 variables were tested. The level of statistical significance using the Bonferroni correction (Bühner and Ziegler, 2009) was adapted to *p* = 0.002 (0.05/28). To acknowledge for variables of no interest (age and gender), *multiple linear regressions* were carried out at the same level of statistical significance (*p* = 0.002). However, only the calculations regarding the group of nurses were of interest.

## Results

### Well-being: group comparisons (nurses vs. non-helping professionals, women vs. men)

*T-tests for independent samples* showed that nursing staff (*M* = 14.36, SD = 5.36) and *non*-helping professionals (*M* = 14.43, SD = 3.31) did not differ statistically significantly in terms of Well-Being (*p* = 0.854).

Gender differences regarding Well-Being (means and standard deviations, value of *p*) are presented in Table 2. Men and women did not differ significantly in terms of Well-Being. However, female nurses differed from male nurses, but not significantly (*p* = 0.059). The effect size of this result is *d* = −0.208, which can be seen as a small effect. The confidence intervals of female nurses (95% CI [13.62, 14.66]) and male nurses (95% CI [14.26, 16.25]) overlapped.

**Table 2:** Gender differences of well-being.

	Women	Men	Female nurses	Male nurses	Female controls	Male controls
Well-being						
Mean (*M*)	14.23	14.82	14.14	15.25	14.40	14.48
Standard deviation (*SD*)	5.38	5.22	5.40	5.10	5.33	5.30
*p*	0.148		**0.059**		0.900	

*Statistical significance level of *p* = 0.05.

For differences (in Number of participants and percentage) in depressive symptoms of nurses vs. *non*-helping professionals and women vs. men, are depicted in Table 3.

**Table 3:** Differences (in Number of participants and percentage) regarding depressive symptoms (WHO-5: score < 13) of nurses vs. controls, women vs. men.

	*n* (total)	*n* (depressive)
Nurses	518	171 (33%)
Controls	335	107 (32%)
Women	620	210 (34%)
Men	233	68 (29%)

### Relationship of personality traits and styles with well-being in nurses

Table 4 shows the correlations of personality traits and styles with Well-Being in the group of nurses and *non*-helping professionals.

**Table 4:** Correlations of personality styles (PS) and personality traits with well-being in the group of nurses and of controls.

	Nurses	Controls
PSSI		
*Willful-paranoid PS*	*r* = −0.369^**^, *p* < 0.001	*r* = −0.428^**^, *p* < 0.001
*Reserved-schizoid PS*	*r* = −0.283^**^, *p* < 0.001	*r* = −0.372^**^, *p* < 0.001
*Foreboding-schizotypical PS*	*r* = −0.017, *p* = 0.705	*r* = −0.072, *p* = 0.190
*Spontaneous-borderline PS*	*r* = −0.514^**^, *p* < 0.001	*r* = −0.568^**^, *p* < 0.001
*Amiable-histrionic PS*	*r* = 0.215^**^, *p* < 0.001	*r* = 0.292^**^, *p* < 0.001
*Ambitious-narcissistic PS*	*r* = −0.104, *p* = 0.018	*r* = −0.005, *p* = 0.929
*Self-critical-self-insecure PS*	*r* = −0.384^**^, *p* < 0.001	*r* = −0.522^**^, *p* < 0.001
*Loyal-dependent PS*	*r* = −0.314^**^, *p* < 0.001	*r* = −0.349^**^, *p* < 0.001
*Carefully-obsessive-compulsive P*	*r* = −0.067, *p* = 0.125	*r* = −0.073, *p* = 0.185
*Critical-negativistic PS*	*r* = −0.414^**^, *p* < 0.001	*r* = −0.455^**^, *p* < 0.001
*Silent-depressive PS*	*r* = −0.601^**^, *p* < 0.001	*r* = −0.653^**^, *p* < 0.001
*Helpful-selfless PS*	*r* = −0.279^**^, *p* < 0.001	*r* = −0.383^**^, *p* < 0.001
*Optimistic-rhapsodic PS*	*r* = 0.348^**^, *p* < 0.001	*r* = 0.472^**^, *p* < 0.001
*Assertive-antisocial PS*	*r* = 0.098, *p* = 0.026	*r* = 0.120, *p* = 0.028
FPI-R		
*Life satisfaction*	*r* = 0.603^**^, *p* < 0.001	*r* = 0.762^**^, *p* < 0.001
*Social orientation*	*r* = 0.013, *p* = 0.771	*r* = 0.090, *p* = 0.099
*Achievement orientation*	*r* = 0.228^**^, *p* < 0.001	*r* = 0.290^**^, *p* < 0.001
*Inhibitedness*	*r* = −0.332**, *p* < 0.001	*r* = −0.414^**^, *p* < 0.001
*Impulsiveness*	*r* = −0.352^**^, *p* < 0.001	*r* = −0.387^**^, *p* < 0.001
*Aggressivenes*	*r* = −0.141^**^, *p* < 0.001	*r* = −0.114, *p* = 0.037
*Strain*	*r* = −0.546^**^, *p* < 0.001	*r* = −0.538^**^, *p* < 0.001
*Somatic complaints*	*r* = −0.435^**^, *p* < 0.001	*r* = −0.545^**^, *p* < 0.001
*Health concern*	*r* = 0.063, *p* = 0.152	*r* = 0.099, *p* = 0.071
*Frankness*	*r* = −0.196^**^, *p* < 0.001	*r* = −0.231^**^, *p* < 0.001
*Extraversion*	*r* = 0.239^**^, *p* < 0.001	*r* = 0.346^**^, *p* < 0.001
*Emotionality*	*r* = −0.566^**^, *p* < 0.001	*r* = −0.639^**^, *p* < 0.001

*Statistical significance level of *p* = 0.002.

Multiple linear regressions were performed in statistically significant correlations between Well-Being and personality traits or styles in nurses. No extreme cases were found among potential outliers, which is why none were excluded and there was no multicollinearity. Normally distributed residuals were expected based on a P-P plot. The predictor *Spontaneous-Borderline Personality Style* was able to predict Well-Being as significant (*p* < 0.001, β = −0.501). The predictors sex (*p* = 0.507) and age (*p* = 0.938) were excluded due to insufficient statistical significance (*p* = 0.120). The model has no auto-correlation as the value of the Durbin-Watson statistic was 2.160. With a multiple determination coefficient (R2) of 0.264 (corrected R2 of 0.263), our model has a strong explanation of the variance. Also the predictor *Silent-depressive Personality Style* was statistically significant (*p* < 0.001) for predicting Well-Being. Due to insufficient statistical significance the predictor sex (*p* = 0.297) and age (*p* = 0.020) were excluded. The model has no auto-correlation as the value of the Durbin-Watson statistic was 2.139. With a multiple determination coefficient (R2) of 0.361 (corrected R2 of 0.359), our model has a moderate explanation of the variance. The predictors *Life Satisfaction* and age were able to predict Wellbeing statistically significant: *F*(2, 515) = 156.467, *p* < 0.001. The predictor sex was excluded due to insufficient statistical significance (*p* = 0.479). The regressions-coefficients age (β = 0.057; *p* = 0.001) and *Life Satisfaction* (β = 1.068; *p* < 0.001) were statistically significant. The model has no auto-correlation as the value of the Durbin-Watson statistic was 2.110. With a multiple determination coefficient (R2) of 0.378 (corrected R2 of 0.376), our model has a moderate explanation of the variance. *Strain* and age were statistically significant predictors of Wellbeing: *F*(2, 515) = 118.897, *p* < 0.001. The predictor sex was excluded due to insufficient statistical significance (*p* = 0.576). The regressions-coefficients age (β = 0.064; *p* < 0.001) and Strain (β = −0.862; *p* < 0.001) were statistically significant. The model has no auto-correlation as the value of the Durbin-Watson statistic was 2.038 and there was no multicollinearity. With a multiple determination coefficient (R2) of 0.316 (corrected R2 of 0.313), our model has a moderate explanation of the variance. Also, the predictor *Emotionality* was a statistically significant (*p* < 0.001, β = 0.057) predictor of Well-Being. The predictor sex was excluded due to insufficient statistical significance (*p* = 0.629), as well as age (*p* = 0.056). The model has no auto-correlation as the value of the Durbin-Watson statistic was 2.060. With a multiple determination coefficient (R2) of 0.320 (corrected R2 of 0.319), our model gives moderate explanation of the variance.

## Discussion

The main conclusions from our results were that nurses did not differ from the control group in their Well-Being. Men and women also did not differ in their Well-Being. The Well-Being of nurses and control subjects correlated roughly with the same personality traits and styles (they differed only in the level of correlations). However, with respect to nurses and Well-Being, the following personality traits and styles correlated most strongly with Well-Being, namely, S*pontaneous-Borderline PS, Silent-Depressive PS, Life Satisfaction, Strain,* and *Emotionality*. We focused only on correlations above 0.5 because we wanted to point out only high correlations for clarity. Regarding Well-Being or depressive symptoms, one-third of nurses, one-third of nonhelping professionals, one-third of men, and one-third of women suffered from low well-being or depressive symptoms during the pandemic.

The COVID-19 pandemic resulted in a 27.6% increase in cases of major depressive disorder worldwide in 2020 (Daly and Robinson, 2022). Regarding Well-Being or depressive symptoms in our study, one-third of nurses and one-third of non-helping professionals suffered from low Well-Being or depressive symptoms during the pandemic. Thus, this finding could be replicated.

### Personality of a person prone to depressive symptoms during COVID (especially nurses)

Most studies on COVID-19 and personality have been conducted using personality dimensions based on the Big Five personality model, such as the findings of Nikčević et al. (2021), who showed that during the COVID-19 pandemic, neuroticism was positively correlated with depressive symptoms and extraversion, agreeableness, conscientiousness, and openness were negatively correlated with depressive symptoms. Other findings suggest that high levels of neuroticism are risky, whereas high levels of extraversion, conscientiousness, or agreeableness are protective against pandemic-related stress (Starcevic and Janca, 2022). Thus, in our study, more personality variables were correlated with well-being. In addition, nurses were specifically considered here.

Our results showed that a nurse who is at risk of suffering from depressive symptoms during a stressful time, for example during COVID-19 pandemic, is characterized by an intense emotionality (*Borderline Personality Style*). This manifests itself on the one hand in a spontaneous enthusiasm for positive things, but on the other hand in an impulsive rejection of things or people who show negative qualities. Also, this person experiences fewer positive feelings, and more negative ones, so these people often feel depressed, worthless, inadequate and pessimistic (*Silent-Depressive Personality Style*). This person is also dissatisfied with the present and former living conditions, partnership and profession. She/He thinks a lot about life and is often dissatisfied with everything and feels unhappy (*Life Satisfaction*). They feel easily stressed when faced with many tasks (*Strain*). Also this person has inner confusion and problems. She/He is quickly irritable, feels tired and exhausted and is prone to mood swings and does not feel properly understood by anyone. Such a person is also nervous and prone to psychosomatic symptoms (*Emotionality*). According to Fahrenberg et al. (2010), *Emotionality* covers essential components of neuroticism. Meta-analytic results highlighted neuroticism as negative Well-Being (Anglim et al., 2020) and a study by Caci et al. (2020) suggested that low neuroticism is crucial in coping with the COVID-19 pandemic, which explains our result. What should also be emphasized is (as can be seen in Table 4) the correlations are not only highly significant, but also often very high. The findings described above (Nikčević et al., 2021; Starcevic and Janca, 2022) that Well-Being is negatively correlated with neuroticism are now also reflected in our results (see *Emotionality*, Table 4). In our results, extraversion correlates positively with well-being, just as in the studies (Nikčević et al., 2021, Starcevic and Janca, 2022), but only in a low correlation (see Table 4).

### Limitations

Our study was cross-sectional rather than longitudinal, so no comparison to pre-pandemic status was possible. Therefore, we could not demonstrate whether the workload of nursing staff changed during the current pandemic, as other studies suggest. Our dropout rate was very high, so helpful, motivated subjects may be overrepresented because they are more willing to participate in a survey without direct reward. In addition, the control group was very inhomogeneous because a variety of occupational groups were found in it. Nurses were also from a wide variety of departments, so this sample was not completely homogeneous either. In addition, only personal well-being was asked, not other criteria that might indicate psychological distress. Other variables that might influence personal well-being (e.g., private life events) were not surveyed, nor was educational status.

### Conclusion

Nurses and *non*-helping professionals did not differ significantly in terms of Well-Being, which means that Well-Being of nurses on the COVID front is no worse than that of other professions (architects, insurance brokers, and craftsmen). The pandemic can, but does not necessarily have to be stressful, depending on personality. However, certain personality variables affected Well-Being similarly in both groups. Consequently, certain personality variables are a stronger predictor of depressive symptoms during the COVID-19 pandemic than occupation, namely, the lower the well-being, the higher the *Spontaneous Borderline PS, Silent Depressive PS*, and *Strain*. However, the higher the *Life Satisfaction* personality trait, the higher the Well-Being.

According to the results, increasing stress tolerance through stress management strategies and therapy to support emotion regulation, e.g., through dialectical-behavioral psychotherapy (Sutor, 2022), could be very important for people who are burdened by low well-being during a crisis, such as a pandemic. People who are at risk of having low well-being in such a crisis, according to our results, also suffer from many inner conflicts and problems and also tend to have psychosomatic symptoms, which is why Psychodynamically Oriented Psychotherapy would be one of the methods that could be offered (Ermann and Waldvogel, 2008). In Table 5, you can see possible psychotherapeutic interventions for these individuals who need psychological support in a crisis like the pandemic. For example, this support could be offered as part of employment through the facility. Through our findings, individuals were identified as being at risk for developing mental health problems during a pandemic. The risk factors for their well-being became clearer through our study results.

**Table 5:** Strategies to deal with symptoms (of people who are at increased risk during COVID-19 pandemic to suffer from low well-being)—based on the book by Sutor (2022).

Symptoms	Strategies	Can be found at
*Intense emotionality*	Emotion regulation strategies	Dialectic behavioral therapy
*Impulsiveness* (e.g.*, an impulsive rejection of things or people who show negative qualities*)	Emotion regulation strategies	Dialectic behavioral therapy
*Quickly irritable and nervous*	Emotion regulation strategies, Stress management strategies	Dialectic behavioral therapy, behavioral therapy
*Feel unhappy*	Find out which thoughts and behaviors are related to these feelings and which maintain these feelings	Behavioral therapy
*Fewer positive feelings*		
*Hence more depressed*		
*Feel worthless*		
*Be pessimistic*		
*Often dissatisfied with the Present and former living conditions*		
*Feel easily stressed when faced with many tasks*	Stress management strategies (e.g., Relaxation procedures)	Behavioral therapy
*Has many inner conflicts and problems, is prone to psychosomatic symptoms*	To explore unconscious reasons	Psychodynamically oriented psychotherapy

### Outlook

Future studies investigating health care professionals’ Well-Being should focus on their personality, because it is crucial to identify people at risk of developing low Well-Being due to their personality factors.

## Data availability statement

The raw data supporting the conclusions of this article will be made available by the authors, without undue reservation.

## Ethics statement

The studies involving humans were approved by Ethics Commission of the State of Salzburg. The studies were conducted in accordance with the local legislation and institutional requirements. The participants provided their written informed consent to participate in this study.

## Author contributions

VM, WA, AK, and MS: conceptualization. VM and AK: data collection and analysis, methodology, and project administration. WA, AK, and MS: supervision and writing—review and editing. VM and MS: writing—original draft. All authors contributed to the article and approved the submitted version.

## Conflict of interest

The authors declare that the research was conducted in the absence of any commercial or financial relationships that could be construed as a potential conflict of interest.

## Publisher’s note

All claims expressed in this article are solely those of the authors and do not necessarily represent those of their affiliated organizations, or those of the publisher, the editors and the reviewers. Any product that may be evaluated in this article, or claim that may be made by its manufacturer, is not guaranteed or endorsed by the publisher.

## References

[ref1] Ambrosetti J. Macheret L. Folliet A. Wullschleger A. Amerio A. Aguglia A.. (2021). Impact of the COVID-19 pandemic on psychiatric admissions to a large Swiss emergency department: an observational study. Int. J. Environ. Res. Public Health 18:1174. doi: 10.3390/ijerph18031174, PMID: 33525740 PMC7908206

[ref2] Amerio A. Lugo A. Stival C. Fanucchi T. Gorini G. Pacifici R.. (2021). COVID-19 lockdown impact on mental health in a large representative sample of Italian adults. J. Affect. Disord. 292, 398–404. doi: 10.1016/j.jad.2021.05.117 34139414 PMC8777065

[ref3] Anglim J. Horwood S. Smillie L. D. Marrero R. J. Wood J. K. (2020). Predicting psychological and subjective well-being from personality: a meta-analysis. Psychol. Bull. 146, 279–323. doi: 10.1037/bul0000226, PMID: 31944795

[ref4] Ashley C. James S. Williams A. Calma K. Mcinnes S. Mursa R.. (2021). The psychological well-being of primary healthcare nurses during COVID-19: a qualitative study. J. Adv. Nurs. 77, 3820–3828. doi: 10.1111/jan.14937, PMID: 34142734 PMC8447215

[ref5] Babić R. Babić M. Rastović P. Ćurlin M. Šimić J. Mandić K.. (2020). Resilience in health and illness. Psychiatr. Danub. 32, 226–232. PMID: 32970640

[ref6] Brähler E. Mühlan H. Albani C. Schmidt S. (2007). Teststatistische prüfung und normierung der deutschen versionen des EUROHIS-QOL Lebensqualität-index und des WHO-5 Wohlbefindens-index [testing and standardization of the German version of the EUROHIS-QOL and WHO-5 quality-of life-indices]. Diagnostica 53, 83–96. doi: 10.1026/0012-1924.53.2.83

[ref7] Bühner M. Ziegler M. (2009). Statistik für Sozialwissenschaftler [Statistics for Social Scientists] München: Pearson.

[ref8] Bundesministerium für Gesundheit [The Federal Ministry of Health] (2021). Nichtärztliches Gesundheitspersonal 2020 in Krankenanstalten nach Geschlecht, Fachrichtung und Bundesland. Available at: file:///C:/Users/Startklar/Downloads/nichtaerztliches_gesundheitspersonal_2020_in_krankenanstalten_nach_geschle.pdf (Accessed December 31, 2020).

[ref9] Caci B. Miceli S. Scrima F. Cardaci M. (2020). Neuroticism and fear of COVID-19. The interplay between boredom, fantasy engagement, and perceived control over time. Front. Psychol. 11:574393. doi: 10.3389/fpsyg.2020.574393, PMID: 33154730 PMC7588354

[ref10] Chidiebere O. E. Tibaldi L. La Torre G. (2020). The impact of COVID-19 pandemic on mental health of nurses. Clin. Ter. 171, 399–400. doi: 10.7417/CT.2020.2247 32901781

[ref11] Daly M. Robinson E. (2022). Depression and anxiety during COVID-19. Lancet 399:518. doi: 10.1016/S0140-6736(22)00187-8, PMID: 35123689 PMC8813060

[ref12] Ermann M. Waldvogel B. (2008). “Psychodynamische Psychotherapie—Grundlagen und klinische Anwendungen” in Psychiatrie und Psychotherapie. eds. Möller H. J. Laux G. Kapfhammer H. P. (Berlin, Heidelberg: Springer).

[ref13] Fahrenberg J. Hampel R. Selg H. (2010). Freiburger Persönlichkeitsinventar (FPI-R): Manual [Freiburg Personality Inventory Revised: Manual] Gottingen: Hogrefe.

[ref14] Galanis P. Vraka I. Fragkou D. Bilali A. Kaitelidou D. (2021). Nurses' burnout and associated risk factors during the COVID-19 pandemic: a systematic review and meta-analysis. J. Adv. Nurs. 77, 3286–3302. doi: 10.1111/jan.14839, PMID: 33764561 PMC8250618

[ref15] Gómez-Ochoa S. A. Franco O. H. Rojas L. Z. Raguindin P. F. Roa-Díaz Z. M. Wyssmann B. M.. (2021). COVID-19 in health-care workers: a living systematic review and Meta-analysis of prevalence, risk factors, clinical characteristics, and outcomes. Am. J. Epidemiol. 190, 161–175. doi: 10.1093/aje/kwaa191, PMID: 32870978 PMC7499478

[ref35] Hossain M. M. Tasnim S. Sultana A. Faizah F. Mazumder H. Zou L.. (2020). Epidemiology of mental health problems in COVID-19: a review. F1000Res 9:636. doi: 10.12688/f1000research.24457.1, PMID: 33093946 PMC7549174

[ref16] IBM Statistics SPSS (2021). [Computer software]. (Version 27) IBM Corp. Available at: https://www.ibm.com/at-de/products/spss-statistics

[ref38] Index der Weltgesundheitsorganisation zum Wohlbefinden WHO-5 (2022). Available at: https://www.prevention-depression.lu/wp-content/uploads/WHO-5.pdf (Accessed August 22, 2022).

[ref17] Kuhl J. Kazén M. (2009). Persönlichkeits-Stil- und Störungs-Inventar (PSSI): Manual [Personality Style and Disorder Inventory (PSSI): Manual] Gottingen: Hogrefe.

[ref18] Labrague L. J. De Los S. J. A. A. (2020). COVID-19 anxiety among front-line nurses: predictive role of organisational support, personal resilience and social support. J. Nurs. Manag. 28, 1653–1661. doi: 10.1111/jonm.13121, PMID: 32770780 PMC7436313

[ref19] Lai J. Ma S. Wang Y. Cai Z. Hu J. Wei N.. (2020). Factors associated with mental health outcomes among health care workers exposed to coronavirus disease 2019. JAMA Netw. Open 3:e203976. doi: 10.1001/jamanetworkopen.2020.3976, PMID: 32202646 PMC7090843

[ref20] Lecic-Tosevski D. Vukovic O. Stepanovic J. (2011). Stress and personality. Psychiatriki 22, 290–297. PMID: 22271841

[ref21] Li Y. Scherer N. Felix L. Kuper H. (2021). Prevalence of depression, anxiety and post-traumatic stress disorder in health care workers during the COVID-19 pandemic: a systematic review and meta-analysis. PLoS One 16:e0246454. doi: 10.1371/journal.pone.0246454, PMID: 33690641 PMC7946321

[ref22] LimeSurvey Computer software. Version 4.3.15. (2020). LimeSurvey GmbH. Available at: https://www.limesurvey.org/de

[ref23] Lu M. Zhang F. Tang X. Wang L. Zan J. Zhu Y.. (2022). Do type a personality and neuroticism moderate the relationships of occupational stressors, job satisfaction and burnout among Chinese older nurses? A cross-sectional survey. BMC Nurs. 21:88. doi: 10.1186/s12912-022-00865-7, PMID: 35428288 PMC9013170

[ref24] Marcomini I. Agus C. Milani L. Sfogliarini R. Bona A. Castagna M. (2021). COVID-19 and post-traumatic stress disorder among nurses: a descriptive cross-sectional study in a COVID hospital. Med. Lav. 112, 241–249. doi: 10.23749/mdl.v112i3.11129, PMID: 34142675 PMC8223933

[ref25] Mason R. Roodenburg J. Williams B. (2020). What personality types dominate among nurses and paramedics: a scoping review? Australas. Emerg. Care 23, 281–290. doi: 10.1016/j.auec.2020.06.001 32694015

[ref26] Nédélec M. Lienhart N. Martinent G. Doron J. (2021). Personality traits, stress appraisals and sleep in young elite athletes: a profile approach. Eur. J. Sport Sci. 21, 1299–1305. doi: 10.1080/17461391.2020.1829716, PMID: 32977726

[ref27] Nikčević A. V. Marino C. Kolubinski D. C. Leach D. Spada M. M. (2021). Modelling the contribution of the big five personality traits, health anxiety, and COVID-19 psychological distress to generalised anxiety and depressive symptoms during the COVID-19 pandemic. J. Affect. Disord. 279, 578–584. doi: 10.1016/j.jad.2020.10.053, PMID: 33152562 PMC7598311

[ref28] Nobari H. Fashi M. Eskandari A. Villafaina S. Murillo-Garcia Á. Pérez-Gómez J. (2021). Effect of COVID-19 on health-related quality of life in adolescents and children: a systematic review. Int. J. Environ. Res. Public Health 18:4563. doi: 10.3390/ijerph18094563, PMID: 33923120 PMC8123423

[ref29] Riedel B. Horen S. R. Reynolds A. Hamidian J. A. (2021). Mental health disorders in nurses during the COVID-19 pandemic: implications and coping strategies. Front. Public Health 9:707358. doi: 10.3389/fpubh.2021.707358, PMID: 34765579 PMC8575697

[ref30] Schlatter S Louisy S., Canada B., Thérond C., Duclos A., Blakeley C., Lehot J.J., Rimmelé T., Guillot A., Lilot M. & Debarnot U. (2022). Personality traits affect anticipatory stress vulnerability and coping effectiveness in occupational critical care situations. Sci. Rep. 12::20965. doi: 10.1038/s41598-022-24905-z, PMID: 36470906 PMC9722917

[ref31] Serrano C. Andreu Y. Murgui S. (2020). The big five and subjective wellbeing: the mediating role of optimism. Psicothema 32, 352–358. doi: 10.7334/psicothema2019.392, PMID: 32711670

[ref32] Sriharan A. West K. J. Almost J. Hamza A. (2021). COVID-19-related occupational burnout and moral distress among nurses: a rapid scoping review. Nurs. Leadersh. 34, 7–19. doi: 10.12927/cjnl.2021.26459, PMID: 33837685

[ref33] Starcevic V. Janca A. (2022). Personality dimensions and disorders and coping with the COVID-19 pandemic. Curr. Opin. Psychiatry 35, 73–77. doi: 10.1097/YCO.0000000000000755, PMID: 34855697 PMC8654116

[ref34] Sutor M. (2022). “Die Dialektisch Behaviorale Therapie (DBT). Neue DBT-orientierte diagnoseübergreifende Konzepte—Schwerpunkt Skills-Training” in ed. Sutor M. Dialectical Behavioral Therapy (DBT). New DBT-Oriented Cross-Diagnosis Concepts—Focus on Skills Training (Heidelberg: Springer)

[ref36] Topp C. W. Østergaard S. D. Søndergaard S. Bech P. (2015). The WHO-5 well-being index: a systematic review of the literature. Psychother. Psychosom. 84, 167–176. doi: 10.1159/000376585 25831962

[ref37] Varghese A. George G. Kondaguli S. V. Naser A. Y. Khakha D. C. Chatterji R. (2021). Decline in the mental health of nurses across the globe during COVID-19: a systematic review and meta-analysis. J. Glob. Health 11:05009. doi: 10.7189/jogh.11.05009, PMID: 33884193 PMC8053406

[ref39] Yayla A. Eskici İ. V. (2021). The relationship of nurses' psychological well-being with their coronaphobia and work-life balance during the COVID-19 pandemic: a cross-sectional study. J. Clin. Nurs. 30, 3153–3162. doi: 10.1111/jocn.15783, PMID: 34337812 PMC8447145

